# Chemoradiation therapy with S-1 for primary squamous cell carcinoma of the rectum: report of three cases

**DOI:** 10.1186/s40792-015-0025-5

**Published:** 2015-02-10

**Authors:** Kimihiko Funahashi, Tetsuo Nemoto, Junichi Koike, Akiharu Kurihara, Hiroyuki Shiokawa, Mistunori Ushigome, Tomoaki Kaneko, Kenichiro Arai, Yasuo Nagashima, Takamaru Koda, Takayuki Suzuki, Satoru Kagami, Yu Suitsu, Hironori Kaneko, Toshikazu Shibuya

**Affiliations:** Department of General and Gastroenterological Surgery, Toho University Medical Center, Omori Hospital, 6-11-1 Omorinishi, Otaku, Tokyo, 143-8541 Japan; Department of Pathology, Toho University Medical Center, Omori Hospital, 6-11-1 Omorinishi, Otaku, Tokyo, 143-8541 Japan

**Keywords:** Squamous cell carcinoma, Rectum, Chemoradiation therapy, S-1

## Abstract

**Purpose:**

Although successful treatment of squamous cell carcinoma (SCC) of the rectum using chemoradiation therapy (CRT) has been reported, a standard protocol has yet to be established. The aim is to ascertain the effectiveness of CRT with S-1 for three patients with SCC of the rectum.

**Case presentation:**

We treated three female patients complaining of rectal bleeding. The patients were diagnosed as having primary SCC of the rectum by means of routine examinations; one of them was a very rare case because of the presence of two lesions in the lower rectum. We treated the patients using CRT with S1 at a radiation dose of 1.8 Gy/fraction given five times weekly (Monday to Friday) to a median dose of 59.4 (45 to 59.4) Gy; S-1 (80 mg/m^2^/day) was administered orally during radiation therapy. One of three patients had an adverse event involving massive hemorrhage from the tumor. All patients exhibited an excellent response to CRT with S-1; two patients had a complete response, and one patient had a partial response and underwent a posterior pelvic exenteration with advancement flap reconstruction as a salvage treatment. Pathological examination of the resected specimen and lymph nodes revealed no tumor cells indicating a pathological complete response. In this series, the response rate was 100%.

**Conclusions:**

We suggest that CRT with S-1 be chosen as the first-line therapy for SCC of the rectum. However, a large study will be required to establish a safe and effective regimen.

## Background

Primary squamous cell carcinoma (SCC) of the anorectum is a very rare disease and there have been few reports regarding SCC in the rectum. Nowadays, chemoradiation therapy (CRT) with 5-fluorouracil (5-FU) plus mitomycin-C (MMC) is the recommended treatment for SCC of the anus based on the findings from a small number of clinical trials [1-3]; surgery is considered as being a salvage treatment. An optimal treatment for primary SCC of the rectum has not been established because of the lack of randomized studies. The choice of the treatment depends on the tumor characteristics, namely, size, location, depth of tumor cell invasion, and distant metastasis. Recently, some studies have reported that CRT might also be effective in the treatment of primary SCC of the rectum [4-9]. Oral fluoropyrimidines including S-1, tegafur-uracil (UFT), and capecitabine have been developed as a therapeutic alternative to the venous infusion of 5-FU. S-1 is an oral anticancer drug composed of tegafur (a prodrug of 5-FU), 5-chloro-2, 4-dihydropyrimidine (CDHP; gimeracil), and potassium oxonate that has the following benefits. It increases the blood 5-FU concentration by inhibiting the metabolism of 5-FU by means of dihydropyrimidine dehydrogenase, and it also enhances the radiation response of colon cancer as compared with 5-FU. In the present case report, we describe three patients with SCC of the rectum that responded to CRT with S-1.

## Case presentation

### Patient 1

**Figure 1:**
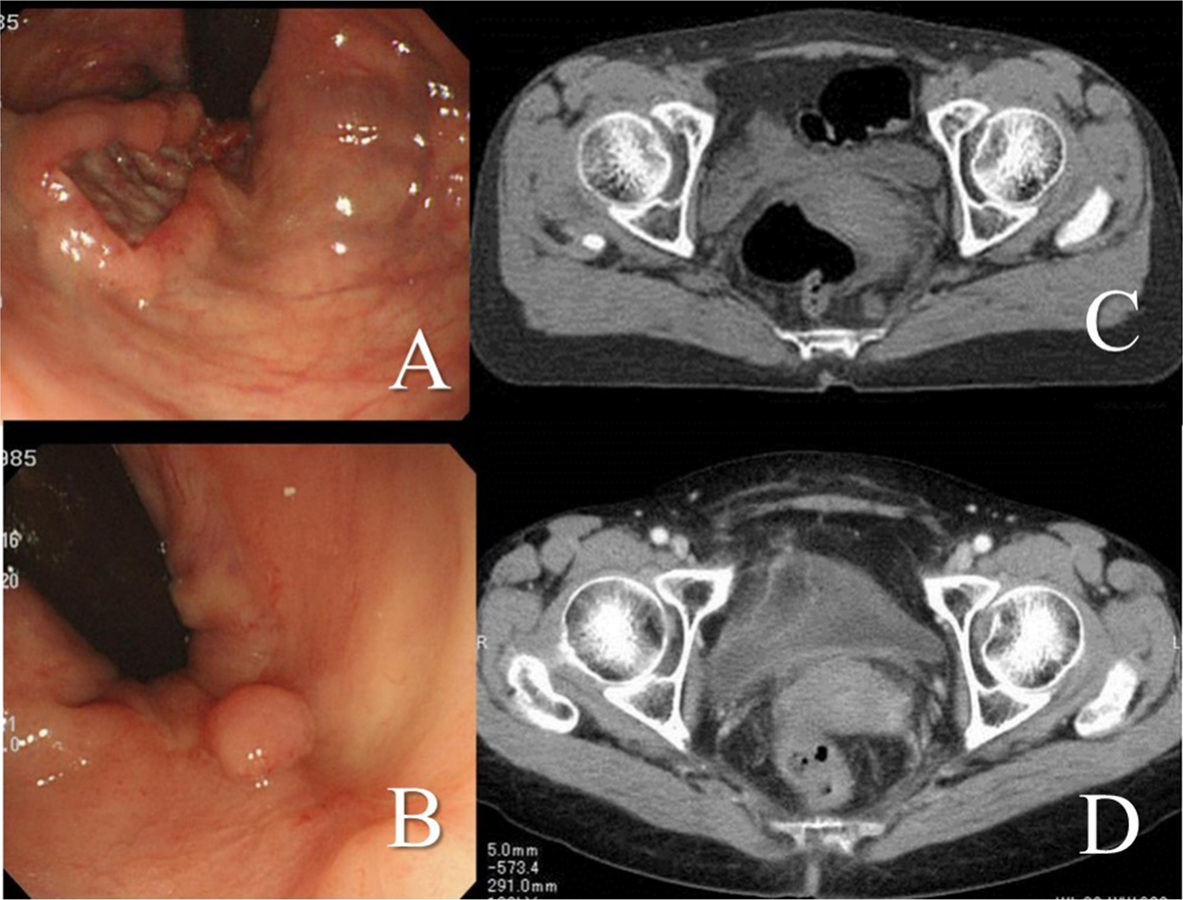
**Effectiveness of chemoradiation therapy with S-1 for patient 1 evaluated by colonoscopy and computed tomography.** Colonoscopy was showing an ulcerative lesion in the lower rectum **(A)**. Lymph node metastasis in the mesorectum was suspected using computed tomography **(C)**. After chemoradiation therapy with S-1, colonoscopy showed a polypoid lesion **(B)**. Biopsies revealed that there were no tumor cells, and lymph node metastasis had disappeared **(D)**.

A 54-year-old woman was diagnosed as having primary SCC of the rectum at another institution. Although abdominoperineal resection (APR) was proposed as a radical treatment at the institution, the patient consulted our institution about alternative treatments including CRT. A colonoscopy revealed the presence of an ulcerative lesion in the lower rectum. Histological analysis of biopsies taken at the time of the colonoscopy revealed SCC. There were no lesions detected at any site other than the rectum using computed tomography (CT) and magnetic resonance imaging (MRI). Lymph node metastasis was found in the mesorectum. Finally, we diagnosed the lesions as primary SCC (cT3 N2a M0, cStage III B) of the rectum. We proposed laparoscopic intersphincteric resection (ISR) for the lesion as a radical treatment, but the patient refused the operation and requested CRT. Therefore, CRT with S-1 was administered. Radiation therapy (RT) at a dose of 1.8 Gy/fraction was given five times weekly (Monday to Friday) to a total dose of 59.4 Gy; S-1 (80 mg/m^2^/day) was administered orally for a total of 55 days (33 days during RT and 22 days alone). The planned CRT with S-1 was achieved without adverse events. The tumor marker, squamous cell carcinoma antigen (SCC Ag), had normalized after CRT with S-1 from a level of 9.3 to 0.9 ng/ml. Colonoscopy revealed the complete response of the lesions, and there were no tumor cells detected in the biopsies taken during colonoscopy (Figure 1). The patient was regularly followed up every 6 months, but there were no findings of recurrence and anorectal dysfunction over a follow-up period of 3 years and 8 months*.*

### Patient 2

**Figure 2:**
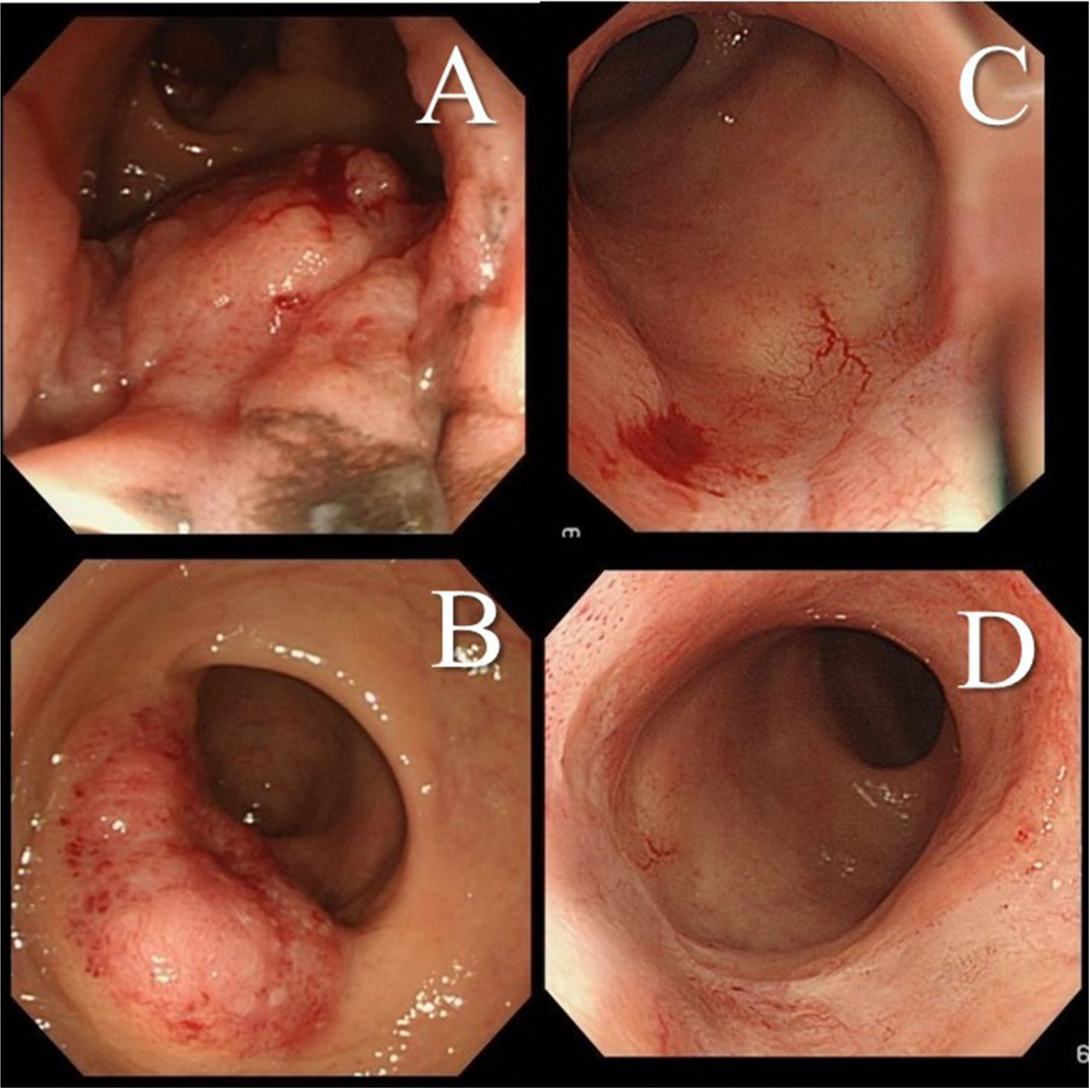
**Colonoscopy findings of patients 2.** Colonoscopy images from patient 2. Two lesions are present in the rectum **(A**, **B)**. No lymph node metastasis was detected using imaging. After radiation therapy at a total dose of 59.4 Gy in combination with S-1, the lesions disappeared **(C**, **D)**. The patient was regularly followed up at 6-month intervals, but no recurrence was found over a follow-up period of 2 years and 1 month.

An 84-year-old woman visited our institution complaining of bloody stools. Digital rectal examination at the outpatients department of our institution revealed a tumor located at about 4 cm from the anal verge. Additionally, a colonoscopy detected another lesion in the lower rectum. Histological evaluation of biopsies taken at the time of the colonoscopy revealed SCC. There were no lesions detected at any other site using CT and MRI. Finally, we diagnosed the lesions as primary SCC of the rectum (cT3 N0 M0, cStage II A). We proposed laparoscopic ISR for these lesions as a radical treatment, but the patient refused the operation because of her advanced age. Consequently, CRT with S-1 was administered for the treatment of these lesions. RT at a dose of 1.8 Gy/fraction was delivered five times weekly (Monday to Friday) to a total dose of 59.4 Gy, and S-1 (80 mg/m^2^/day) was given orally for a total of 50 days (33 days during radiation therapy and 17 days alone). The planned CRT with S-1 was achieved safely without adverse events. The tumor marker, SCC Ag, had normalized after CRT with S-1 from a level of 6.8 to 0.9 ng/ml. Colonoscopy showed a complete response for both lesions, and there were no tumor cells in biopsies taken in the colonoscopy (Figure 2). The patient was regularly followed up at 6-month intervals, and there were no findings of recurrence and anorectal dysfunction over a follow-up period of 2 years and 1 month.

### Patient 3

**Figure 3:**
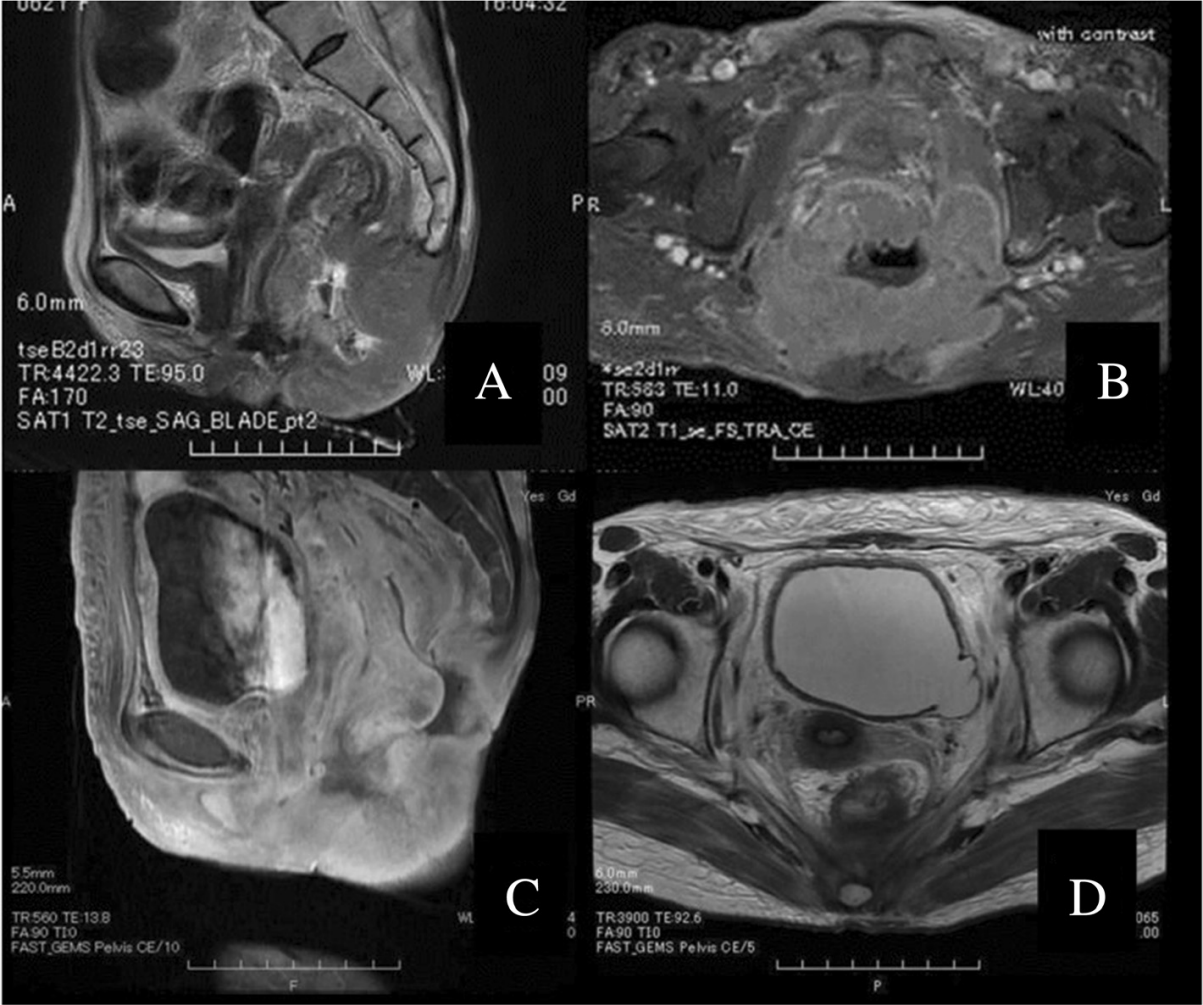
**Magnetic resonance imaging of patient 3.** Magnetic resonance image of patient 3 with squamous cell carcinoma of the rectum **(A**, **B)**. The tumor was diagnosed as being at clinical stage T4b N2b M0. The patient received chemoradiation therapy with S-1 **(C**, **D)**, and then, a posterior pelvic exenteration with advancement flap reconstruction was performed as salvage therapy. Pathological findings revealed no tumor cells in either the resected specimen or the lymph nodes (i.e., a pathological complete response).

A 64-year-old woman visited our institution complaining of analgia. We suspected rectal cancer from the findings of digital examination and immediately hospitalized the patient to observe the lesion and perform open biopsy under spinal anesthesia. It was found that the tumor in the lower rectum had massively invaded the perineum but there were no findings including anal involvement and fistulous tract lined by rectal tumor. Histological analysis of biopsies revealed SCC. Additionally, MRI showed invasion of the levator ani and the vagina (T4b*),* with lymph node metastases involvement including the inguinal lymph nodes. However, there was no distant metastasis*.* We diagnosed the lesion as primary SCC of the rectum*.* The SCC was staged as cT4b N2b M0, cStage III C using the TNM classification. CRT with S-1 for the locally advanced lesion was planned as a primary treatment. RT at a dose of 1.8 Gy/fraction was delivered five times weekly (Monday to Friday) to a total dose of 59.4 Gy*,* and S-1 (80 mg/m2/day) was given orally. However, during treatment*,* the patient developed a massive hemorrhage from the primary lesion and required a blood transfusion. The adverse event was considered as grade 3 in the common terminology criteria for adverse events version 4.0. Finally*,* RT of a total dose of 45 Gy and S-1 (80 mg/m2/day) for a total of 30 days were performed*.* After CRT with S-1, the level of SCC Ag had decreased from a level of 54.3 to 1.2 ng/ml. Evaluation using MRI indicated that the size of the primary lesion had decreased (Figure 3). Consequently, we performed a posterior exenteration with advancement flap reconstruction as salvage therapy. Pathological findings revealed no tumor cells in either the resected specimen or the lymph nodes. Therefore, the patient was judged to have achieved a pathological complete response. After leaving the hospital, the patient was followed up regularly at our outpatient department. She complained of lumbar pain at 1 year after treatment. MRI of the spine showed bone metastasis. RT was administered to palliate the lumbar pain induced by bone metastasis. Finally, the patient died of bone and liver metastases at 1 year and 2 months after surgery.

## Discussion

We treated three patients with SCC of the rectum in our institution between 2000 and 2013 (Table 1). The therapy for SCC of the rectum was approved by the Toho University Medical Center Omori Hospital Institutional Review Board (No. 26-231). All of the patients were female and complained of rectal bleeding. Colonoscopy revealed a tumor located in the lower rectum, and histological analysis of biopsies taken at the time of colonoscopy indicated SCC. CT and MRI examinations did not detect lesions at any other site. Finally, the lesions were diagnosed as primary SCC of the rectum. The occurrence of SCC in the colon and rectum is much rarer than in that the anus. In particular, the occurrence of two lesions in the rectum is extremely rare. The mechanism of the development of SCC in the rectum remains elusive. Human papillomavirus (HPV) has been associated with many squamous cell carcinomas such as oral, vaginal, esophageal, and anal cancer and so on. It is possible that SCC of the rectum is associated with HPV infection as well, but there are no firm evidences for a cause or relationship between infection with HPV and SCC of the rectum [10,11]. In addition, several cases have been reported in patients with ulcerative colitis [12-15] and in patients with infections including Schistosomiasis [16] and *Entamoeba histolytica* [17].

**Table 1:** **Patients treated with chemoradiation therapy with S-1**

**Number**	**Gender**	**Age**	**Location**	**Size (mm)**	**cStage**	**RT (gray/fraction)**	**CT**	**Adverse events (CTCAE v4.0)**	**SCC Ag before CRT**	**SCC Ag after CRT**	**Operation**	**Response**	**Prognosis**
1	F	54	Rb	21.5	III B	59.4/33	S-1 alone	None	9.3	0.9	-	CR	Disease-free for 3 years 8 months
2	F	84	Rb	42.6	II A	59.4/33	S-1 alone	None	6.8	0.9	-	CR	Disease-free for 2 years 1 month
3	F	62	Rb-a	44.4	III C	45/25	S-1 alone	Hemorrhage (G-3)	54.3	1.2	PPE	pCR	Bone and liver metastasis at 1 year 2 months
			Rb	125									

RT, radiation therapy; CT, chemotherapy; CTCAE*,* common terminology criteria for adverse events*;* SCC Ag, squamous cell carcinoma antigen; CRT, chemoradiation therapy; Ra, rectum above the peritoneal reflection; Rb, rectum below the peritoneal reflection; CR, complete response; PPE, posterior pelvic exenteration; pCR, pathological complete response.

Currently, treatment for SCC of the rectum depends on tumor size, location, depth of invasion, lymph node involvement, and the presence of distant organ metastasis. Local excision is appropriate for T1 or selected T2 tumor, and low anterior resection or abdominoperineal resection is required for advanced tumor. Dyson et al. [18], however, reported that prognosis for patients with SCC of the rectum who received surgical treatment was poor. Surgical treatment also accompanies some problems such as surgical site infection, leakage, and urinary/sexual complications. Some researchers have reported several cases treated using CRT as the primary therapy or in conjunction with surgery [14,15,19-24]. Recently, Clark et al. [4], Rasheed et al. [5], and Sturgeon et al. [25] reported the success of CRT in the treatment of SCC of the rectum. Their regimens involved primary 5-FU-based treatment together with either MMC or cisplatin, which were the same regimens as used for SCC of the anus. Additionally, some experimental treatments using CRT with S-1 + cisplatin, S-1 + MMC, and S-1 alone for SCC of the anus have been reported in Japanese studies [26-34]. These studies found that all patients treated using CRT with S-1 alone had an excellent response to treatment (Table 2). S-1 is an oral anticancer drug that has the following benefits. S-1 increases the blood 5-FU concentration by inhibiting the metabolism of 5-FU by means of dihydropyrimidine dehydrogenase (DPD) and shows significant effectiveness to adenocarcinoma with high-DPD activity of the stomach [35-37], pancreas [38,39], biliary tract [40-42], and so on [43,44]*.* Moreover*,* S-1 is more effective as a tumor radiation response enhancer than 5-FU [45,46]. In reports by Vezeridis et al. [47] and Maritinez-Gonzalez et al. [20], disease control for SCC of the rectum treated using 5-FU and RT was not sufficient. In our series, RT was delivered at a total dose of 59.4 Gy in 1.8 Gy/fraction 5 days weekly (Monday to Friday) over a period of 33 days, and S-1 (80 mg/m^2^/day) was given orally during RT. Patients 1 and 2 were able to safely undergo treatment with S-1, whereas patient 3 was not. Regarding the effectiveness of treatment, all patients had an excellent tumor response; there was a complete response in two patients and a partial response in one. Although patient 3 required a posterior pelvic exenteration with advancement flap reconstruction as a salvage therapy, pathological findings revealed no tumor cells in either the resected specimen or the lymph nodes. Therefore, the response rate was 100% in this series. Phan et al. [48] and Lukan et al. [49] have reported on the treatment of refractory SCC of the anus using cetuximab. Molecular-targeted therapy such as cetuximab for SCC with an epithelial growth factor receptor might be useful in the treatment of a refractory SCC, such as that seen in patient 3.

**Table 2:** **Reported cases of squamous cell carcinoma of the anus treated chemoradiation therapy with S-1**

**Author**		**Gender**	**Age**	**Location**	**cStage**	**RT (Gy)**	**Chemotherapy**	**Response**	**Prognosis**
Miyamoto S et al. [26]	2009	F	61	AC	IV	30	S-1 + CDDP	CR	Disease-free for 20 months
Kuga Y et al. [27]	2009	F	76	AC	III	50	S-1 + CDDP	CR	NR
Shiozawa M et al. [28]	2010	F	48	AC	III	60	S-1 + MMC	CR	Disease-free for 2 years 7 months
		F	65	AC	III	55.8	S-1 + MMC	CR	Disease-free for 2 years 4 months
		F	71	AC	III	58.0	S-1 + MMC	CR	Disease-free for 8 months
Hata T et al. [29]	2011	F	53	AC	I	45	S-1 alone	CR	NR
Baba H et al. [30]	2011	F	79	AC	III	66	S-1 alone	CR	Disease-free for 10 months
Nitori N et al. [31]	2011	F	58	AC	IV	30	S-1 + CDDP	NR	Dead at 16 months
Kuroda M et al. [32]	2012	F	81	AC	IV	50.4	S-1 + CDDP	CR	Disease-free for 1 year 3 months
Sato H et al. [33]	2012	F	83	AC	II	60	S-1 alone	CR	Local recurrence at 9 months and dead at 15 months after CRT
Murata K et al. [34]	2012	F	76	AC	II	60	S-1 alone	CR	Recurrence at 2 years

RT, radiation therapy; AC, anal canal; CR, complete response; NR, not reported.

## Conclusions

The results suggested that CRT with S-1 was chosen as the first-line therapy for SCC of the rectum; although, surgery might be an effective salvage therapy. Evaluation of this modality in a large series of patients with SCC of the rectum will be required to establish safety and efficacy.

## Consent

Informed consent was obtained from the patients for publication of this case report and any accompanying images. A copy of the written consent is available for review by the Editor-in-Chief of this journal.

## Abbreviations

5-FU: 5-Fluorouracil
APR: abdominoperineal resection
CRT: chemoradiation therapy
CT: computed tomography
DPD: dihydropyrimidine dehydrogenase
HPV: human papillomavirus
ISR: intersphincteric resection
MMC: mitomycin-C
MRI: magnetic resonance imaging
RT: radiation therapy
SCC: squamous cell carcinoma
SCC Ag: Squamous cell carcinoma antigen
